# A systematic review and meta-analysis quantifying schistosomiasis infection burden in pre-school aged children (PreSAC) in sub-Saharan Africa for the period 2000–2020

**DOI:** 10.1371/journal.pone.0244695

**Published:** 2020-12-29

**Authors:** Chester Kalinda, Tafadzwa Mindu, Moses John Chimbari

**Affiliations:** 1 University of Namibia, Katima Mulilo, Namibia; 2 Department of Public Health, College of Health Sciences, School of Nursing and Public Health, University of KwaZulu-Natal, Durban, South Africa; Dokkyo Medical University, JAPAN

## Abstract

**Introduction:**

Following the adoption of the World Health Assembly Resolution WHA 65.21 and Neglected Tropical Diseases road map 2021–2030, schistosomiasis control programmes have shifted from morbidity control to disease elimination. However, several gaps continue to be observed in the implementation of control programmes with certain age groups omitted from these campaigns increasing health inequalities and risks of reinfections to previously treated groups. We used the Inverse Variance Heterogeneity (IVhet) model to estimate the prevalence of schistosomiasis infection among preschool-aged children.

**Methods:**

We did a systematic review of peer-reviewed literature on schistosomiasis in sub-Saharan Africa for the period January 1, 2000 to November 30, 2020. Quantitative data for cases of schistosomiasis infection were extracted, including country and region where the studies were done, year of publication and specific schistosome species observed. The IVhet model was used to estimate the pooled prevalence estimate (PPE), the heterogeneity and publication bias.

**Results:**

We screened 2601 articles to obtain 47 eligible studies containing quantitative data on preschool-aged children. Of the selected studies, 44.7% (n = 22) were from East Africa while the least number of studies obtained (2.1%, n = 1) was from Central Africa. 21712 subjects were screened for infection due to *Schistosoma* spp; 13924 for *S*. *mansoni* and 7788 for *S*. *haematobium*. The PPE for schistosomiasis among PreSAC was 19% (95% CI: 11–28). Infection due to *S*. *mansoni* (IVhet PPE: 22% (95% CI: 9–36) was higher than that due to *S*. *haematobium* (15%; 95% CI: 6–25). A Luis Furuya–Kanamori index of 1.83 indicated a lack of publication bias. High level of heterogeneity was observed (I2 > 90%) and this could not be reduced through subgroup analysis.

**Conclusion:**

Schistosomiasis infection among pre-school aged children 6 years old and below is high. This indicates the importance of including this age group in treatment programmes to reduce infection prevalence and long-term morbidities associated with prolonged schistosome infection.

## Introduction

Schistosomiasis is one of the most common parasitic infections caused by blood fluke trematodes and mainly affects poor and marginalized communities with inadequate sanitation and health services [1]. In terms of the number of people affected and at risk of infection, schistosomiasis has been ranked second to malaria [2]. Infection with *Schistosoma* parasites reduces household income and economic productivity [3] and if left untreated, long term morbidity may occur [4–6]. Control of schistosomiasis is a fundamental part of the Sustainable Development Goal (SDG) 3 which seeks to ensure healthy lives and promote well being for all ages with the health target 3.3 in part focusing on ending neglected tropical diseases by 2030, thus contributing to the achievement of universal health coverage (Health target 3.8). Achieving these targets in developing countries is dependent on supporting the research and development of vaccines and medicines, early warning, risk reduction and management of health risks (Target 3b) [7, 8].

Efforts to control schistosomiasis are ongoing through various global and national disease monitoring and control initiatives [9, 10]. These programmes have often been based on school mass drug administration (MDA) using praziquantel. The success indicator has been based on treatment coverage of 75% among school-aged children (SAC) [11, 12] with the inclusion of adults in high-risk settings where disease prevalence in SAC is 50% [12]. Schistosomiasis infection among SAC has often been used as a proxy to determine endemicity and forms the basis for disease control [12, 13] and this has been justified by several school-based studies that observed high prevalence and intensity rates among enrolled SAC. Furthermore, the use of school-based schistosomiasis control programmes in several settings has been cost-effective because of availability ofalready existing infrastructure [14].

However, the success of school-based MDA programmes is highly dependent on the number of SAC enrolled [15] and this usually excludes unenrolled children, those absent from school at the time of implementation [16], preschool-aged children (PreSAC), and certain adolescents and adults [17]. Although there is evidence of schistosomiasis infection among PreSAC, this age group has not been included in many MDA programmes [17]. This increases health inequalities and accumulation of potentially irreversible morbidities due to prolonged infection that ultimately compromises the future wellbeing of children that are not treated early [18]. To design cost-effective monitoring and control activities for schistosomiasis, determination of infection prevalence even among PreSAC is a critical component. We, therefore, used the inverse variance heterogeneity (IVhet) model to estimate the prevalence of schistosomiasis infection among PreSAC in sub-Saharan Africathus illuminate the importance of their inclusion in MDA programmes.

## Methods

### Search strategy and selection criteria

We searched for literature using the Preferred Reporting Items for Systematic Reviews and Meta-Analyses (PRISMA) guidelines [19] in PubMed, MEDLINE, EMBASE and Web of Science databases. Literature searched was based on paediatric schistosomiasis published between January 1, 2000, and November 30, 2020. A broad search strategy combining several terms and restricted to SSA was used. The current study used the search terms “schistosomiasis AND pre-school" OR "pre-school" OR "under five" AND "sub-Saharan Africa" NOT "school-age children" (S1 File). We included studies that were published as peer-reviewed journals, reports and book chapters. In addition, we identified more relevant articles from reference lists of already identified articles. Two reviewers (TM and CK) independently screened through the titles and abstracts to identify relevant reports. Any disagreements were resolved by discussion unless otherwise arbitrated by the third author (MJC). The inclusion criteria for all articles were:

- Articles reporting data from any SSA country
- Articles reporting prevalence rates among PreSAC
- Articles that included data on the following outcome of interest: specific schistosome studied, sample size and number of positive cases, age groups of respondents.
- Articles reporting the age of participants to be between 0–5 years old.

### Data abstracted and quality appraisal

Data extracted from the reviewed papers included the first author’s name, year of publication, study country, the region in Africa, sample size, number of cases and age range. The quality of all studies included was assessed using the Joanna Briggs Institute Prevalence Critical Appraisal Tool [20]. Each selected study was assessed using 10 quality control items and for each item fulfilled, a score of 1 was given while a 0 was given for each unfulfilled item. An aggregate of all the scores was generated and converted into an index. Based on the quality indices generated, studies were classified as having low (0.0–0.3), moderate (0.4–0.6) or high (0.7–1.0) quality (S2 File).

### Data analysis

We used the inverse variance heterogeneity (IVhet) model [21] in MetaXL to obtain the Pooled prevalence estimates (PPE) for the selected studies. Compared to the fixed effect (FE) or random effect (RE) models, the IVhet model regardless of heterogeneity, maintains a correct coverage probability at a lower detected variance [21]. This ensures no underestimation of the statistical error and it maintains modest estimates compared to the RE model. The level of heterogeneity was evaluated using Cochran’s Q statistic and *I*^*2*^ while publication bias was assessed using the Luis Furuya–Kanamori (LFK) index of the Doi plot [22]. We determined the symmetry of the Doi plots using the LFK index. An LFK index shows the level of publication bias depending on the magnitude of the index. An LFK value in the range of ‘±1’ was considered as ‘symmetrical’ and the level of bias classified as “absence of publication bias”. On the other hand, an index value of ‘±2’ was considered as minor asymmetry and classified as “low publication bias” while an index value outside the range of ‘±2’ was classified as major asymmetry and “high publication bias” [22]. Forest plots were used to display the estimated prevalence and their 95% confidence interval. To explore heterogeneity and factors that could potentially influence the observed PPE, we implemented subgroup analysis by stratifying our data according to parasite species and the regions (West Africa, Central, East Africa or Southern Africa) where the studies were conducted; thus assessing heterogeneity between subgroup and within-group.

## Results

### Search results

**Fig 1:**
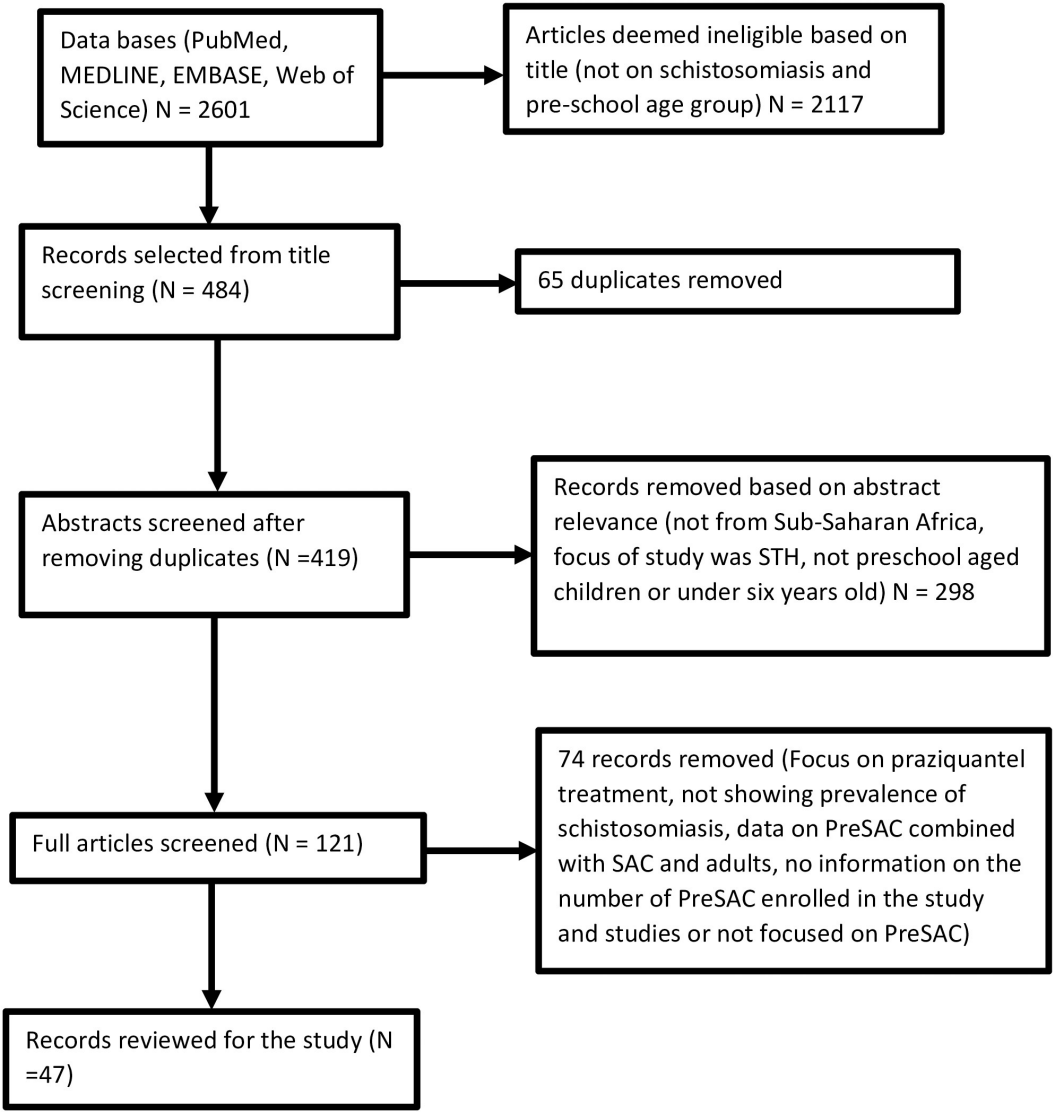
PRISMA flow of study selection.

Fig 1 summarizes the selection process that was used following the PRISMA guidelines. The initial search yielded 2601 studies. After removing duplicates and studies that were deemed irrelevant, following the set inclusion criteria, a total of 47 studies were selected for meta-analysis (Fig 1).

### Study characteristics and PPE analysis

Forty-seven studies were eligible for final inclusion in the study. These studies were conducted in sixteen countries; 4% [23] were from Central Africa (Cameroon), 22% [24–33] were from Southern Africa (Angola, eSwatini, Malawi, South Africa and Zimbabwe), 31% [34–48] were from West Africa (Cote d’Ivoire, Ghana, Mali, Niger, Nigeria and Sierra Leone) and 43% [49–69] were from East Africa (Ethiopia, Kenya, Rwanda, Tanzania and Uganda). Furthermore, 64.1% of the subjects were screened for morbidity outcomes due to *S*. *mansoni* while 35.9% were screened for *S*. *haematobium*. Overall, the studies enrolled a total of 21712 PreSAC.

A wide range of diagnostic tests was used to detect the presence of schistosome ova in urine and stool samples (Table 1). Kato Katz was the commonly used diagnostic test for studies screening for *S*. *mansoni* infection (96.6%, n = 28/29) with one study using the Sodium acetate-acetic acid-formalin (SAF) solution concentration method alone. Other studies used a combination of Kato Katz with SAF (n = 3), Kato Katz and point-of-care test circulating cathodic antigen (POC-CCA) (n = 10), Kato Katz and enzyme-linked immunosorbent assays (ELISAs) (n = 4) and real-time polymerase chain reaction (qPCR) (n = 1). On the other hand, the urine filtration method was widely used among those studies screening for *S*. *haematobium* (95.6%, 22/23) while one study used micro-hematuria in urine measured using urine reagent strips as a proxy-diagnosis for *S*. *haematobium*. Some studies (n = 6) also used a combination of urine filtration methods and the dipstick assay for the diagnosis of *S*. *haematobium* (Table 1).

**Table 1:** List of studies included and diagnostics tests used.

Author name and Year	Sample size	Positive cases	Schistosome species	Country	Regions	Technique	Sampling strategy
Macklina et al. (2018)	21	2	*S*. *haematobium*	Cameroon	Central Africa	Urine filtration	
Chimponda and Mduluza (2020)	145	31	*S*. *haematobium*	Zimbabwe	Southern Africa	Urine filtration	Simple random
Chu et al. (2010)	59	9	*S*. *haematobium*	ESwatini	Southern Africa	Urine filtration	Simple random
Moyo et al. (2016)	143	19	*S*. *haematobium*	Malawi	Southern Africa	Urine filtration	Simple random
Mutsaka-Makuvaza et al. (2018)	535	71	*S*. *haematobium*	Zimbabwe	Southern Africa	Urine filtration	Simple random
Osakunor et al. (2018)	1502	128	*S*. *haematobium*	Zimbabwe	Southern Africa	Urine filtration	Systematic sampling
Sousa-Figueiredo et al. (2012)	1237	124	*S*. *haematobium*	Angola	Southern Africa	Urine strips	Simple random
Mduluza-Jokonya et al. (2020)	415	145	*S*. *haematobium*	Zimbabwe	Southern Africa	Urine filtration	Simple random
Poole et al. (2014)	208	103	*S*. *mansoni*	Malawi	Southern Africa	Kato Katz, POC CCA, ELISA	Semi-random
Sacolo-Gwebu et al. (2019) [1]	998	9	*S*. *haematobium*	South Africa	Southern Africa	Urine filtration	Systematic random sampling
Sacolo-Gwebu et al. (2019) [2]	1143	11	*S*. *mansoni*	South Africa	Southern Africa	Kato Katz	Systematic random sampling
Wami et al. (2015)	104	14	*S*. *haematobium*	Zimbabwe	Southern Africa	Urine filtration, SEA ELISA, Reagent strips	Systematic random sampling
Armoo et al. (2020)	190	48	*S*. *mansoni*	Ghana	West Africa	urine-CCA, real-time PCR, Kato-Katz	Systematic random sampling
Garba et al. (2010) [1]	185	81	*S*. *mansoni*	Niger	West Africa	Kato-Katz	No sampling. All eligible participants included
Garba et al. (2010) [2]	282	161	*S*. *haematobium*	Niger	West Africa	Urine filtration	No sampling. All eligible participants included
Adeniran et al. (2017) [1]	167	6	*S*. *mansoni*	Nigeria	West Africa	Sodium acetate acetic-acid formalin solution	Not specified
Adeniran et al. (2017) [2]	167	8	*S*. *haematobium*	Nigeria	West Africa	Urine filtration, Reagent strip	Not specified
Babatunde et al. (2013)	72	16	*S*. *haematobium*	Nigeria	West Africa	Urine filtration	Random sampling
Bosompem et al. (2004) [1]	80	9	*S*. *haematobium*	Ghana	West Africa	ELISA, Urine filtration, Reagent strip	Not mentioned
Bosompem et al. (2004) [2]	75	0	*S*. *mansoni*	Ghana	West Africa	Kato Katz	Not mentioned
Coulibaly et al. (2013) [1]	242	26	*S*. *haematobium*	Ivory Coast	West Africa	Urine filtration, POC CCA	All eligible children included in the study
Coulibaly et al. (2013) [2]	242	56	*S*. *mansoni*	Ivory Coast	West Africa	Kato Katz	All eligible children included in the study
Dabo et al. (2011)	338	173	*S*. *haematobium*	Mali	West Africa	Urine filtration	No sampling was done due to the small population size
Ekpo et al. (2010)	167	97	*S*. *haematobium*	Nigeria	West Africa	Urine filtration	No sampling was done due to the small number of PSAC
Ekpo et al. (2012a)	83	14	*S*. *haematobium*	Nigeria	West Africa	Urine filtration	No sampling was done due to the small number of PSAC
Ekpo et al. (2012b)	86	45	*S*. *haematobium*	Nigeria	West Africa	Urine filtration, Urine dipstick	No sampling was done due to the small number of PSAC
Hodges et al. (2012)	1803	202	*S*. *mansoni*	Sierra leone	West Africa	Kato Katz	Random selection
Houmsou et al. (2016)	358	63	*S*. *haematobium*	Nigeria	West Africa	Urine filtration	Simple random
Mafiana et al. (2003)	209	150	*S*. *haematobium*	Nigeria	West Africa	Urine filtration	All eligible children
Opara et al. (2007)	126	25	*S*. *haematobium*	Nigeria	West Africa	Urine filtration, dipsticks	All eligible children
Salawu and Alexander (2013)	419	41	*S*. *haematobium*	Nigeria	West Africa	Urine filtration, reagent strips	Random selection
Alemu et al. (2015)	400	1	*S*. *mansoni*	Ethiopia	East Africa	Kato Katz	Not specified
Alemu et al. (2016)	401	45	*S*. *mansoni*	Ethiopia	East Africa	Kato Katz	Two-stage cluster sampling
Lewetegn et al. (2019)	214	9	*S*. *mansoni*	Ethiopia	East Africa	Kato Katz	Not mentioned
Betson et al. (2010)	1295	352	*S*. *mansoni*	Uganda	East Africa	ELISA, Kato Katz	Not mentioned
Pinot de Moira et al. (2013)	426	179	*S*. *mansoni*	Uganda	East Africa	Kato Katz	Not mentioned
G/hiwot et al. (2014)	374	33	*S*. *mansoni*	Ethiopia	East Africa	SAF, Kato Katz	Systematic
Mueller et al. (2019)	71	39	*S*. *mansoni*	Tanzania	East Africa	POC CCA, Kato Katz	All eligible for participation
Nalugwa et al. (2015)	3058	1203	*S*. *mansoni*	Uganda	East Africa	Kato-Katz	Random cluster sampling
Ndokeji et al. (2016)	71	43	*S*. *mansoni*	Tanzania	East Africa	Kato-Katz	Random sampling
Niyituma et al. (2017)	248	42	*S*. *mansoni*	Rwanda	East Africa	Kato Katz, POC CCA	Random sampling
Odogwu et al. (2006)	136	9	*S*. *mansoni*	Uganda	East Africa	Kato Katz, POC CCA, SAF	Systematic random
Ruganuza et al. (2015)	383	170	*S*. *mansoni*	Tanzania	East Africa	Kato Katz, POC CCA	Systematic sampling
Rujeni et al. (2019)	211	20	*S*. *mansoni*	Rwanda	East Africa	Kato Katz, POC CCA	Convenient sampling
Sakari et al. (2017)	361	18	*S*. *mansoni*	Kenya	East Africa	Kato Katz, SAF	Random sampling
Stothard et al. (2011a) [1]	247	19	*S*. *mansoni*	Uganda	East Africa	Kato Katz, POC CCA, ELISA	Not mentioned
Stothard et al. (2011b) [2]	242	115	*S*. *mansoni*	Uganda	East Africa	Kato Katz	All willing to participation
Verani et al. (2011)	216	79	*S*. *mansoni*	Kenya	East Africa	Kato Katz, POC CCA, ELISA	Not mentioned
Masaku et al. (2020)	653	77	*S*. *mansoni*	Kenya	East Africa	Kato-Katz	Simple random
Sassa et al. (2020)	305	11	*S*. *mansoni*	Kenya	East Africa	Kato Katz, POC CCA	Simple random
Kemal et al. (2019)	236	59	*S*. *mansoni*	Ethiopia	East Africa	Kato-Katz	Systematic
Sousa-Figueiredo et al. (2010)	363	225	*S*. *mansoni*	Uganda	East Africa	Kato Katz, SEA-ELISA, CCA	Random sampling

**Fig 2:**
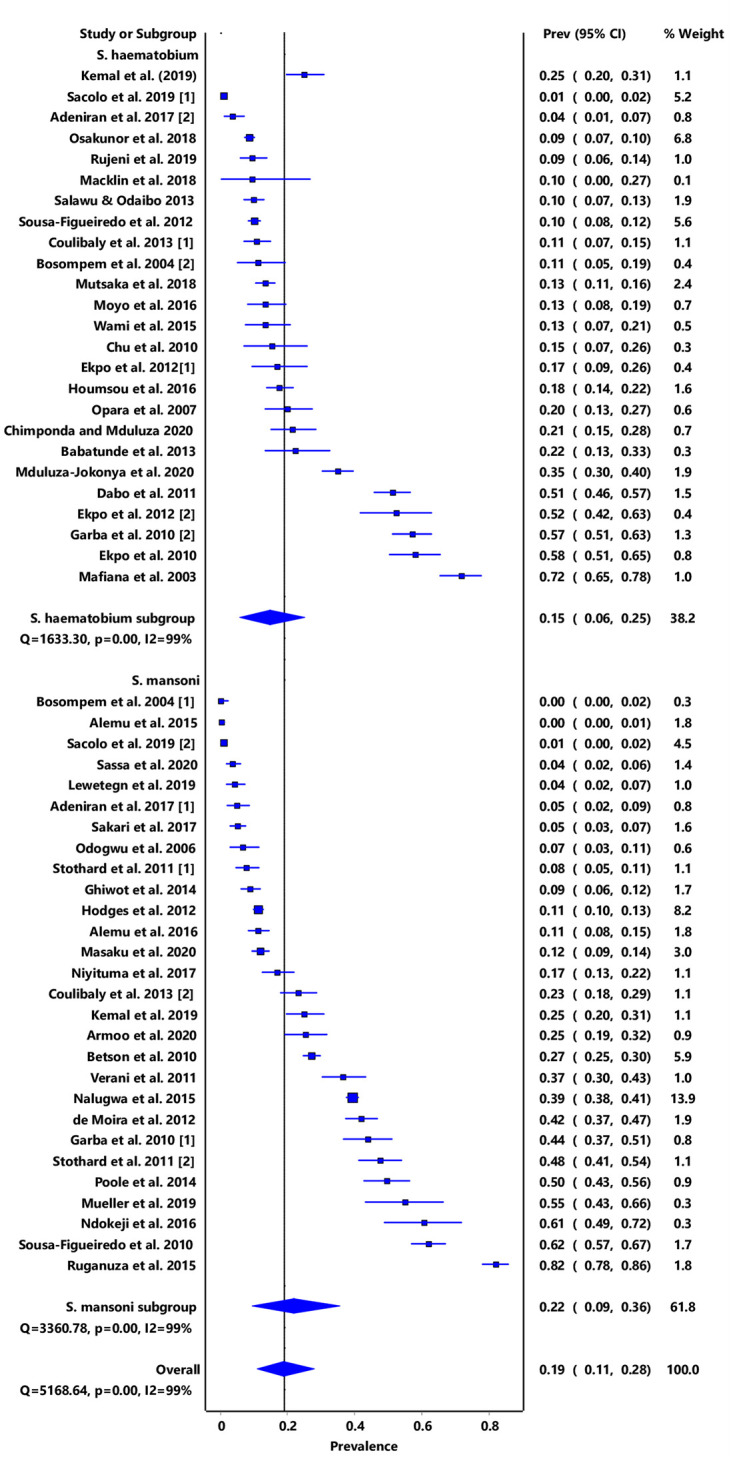
Forest plot of subgroup PPE analysis for schistosome species.

In the overall analysis, the IVhet model showed a pooled prevalence estimate (PPE) of 19% (95% CI: 11–28), with a high degree of heterogeneity (I2 = 99%, *p* < 0.001). Subgroup analysis stratified by schistosome species showed that infection due to *S*. *mansoni* was 22% (95% CI: 9–36) while that due to *S*. *haematobium* was 15% (95% CI: 6–25) (Fig 2).

**Fig 3:**
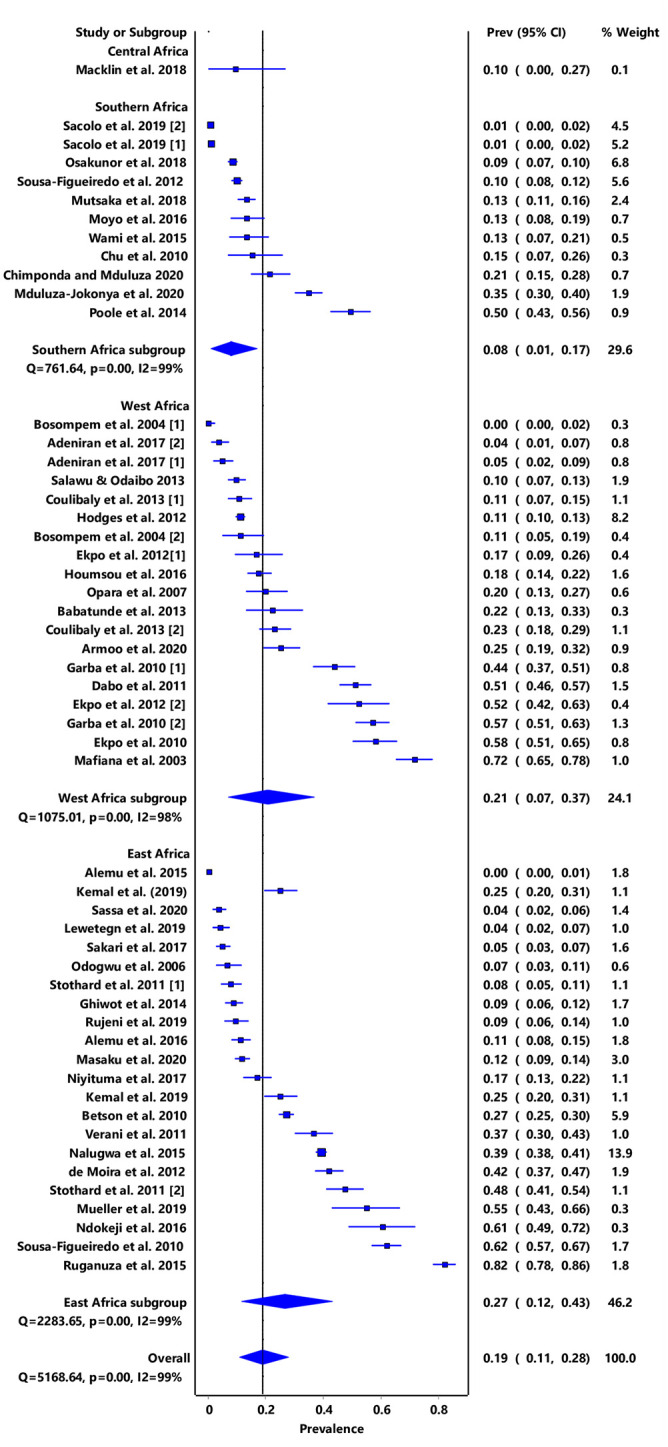
Forest plot of subgrouped PPE analysis for infection prevalence in regions within sub-Saharan Africa.

Furthermore, stratification of infection by region showed that East Africa had a combined PPE of 27% (95% CI: 12–43) while only one study was obtained from Central Africa and had a PPE of 10% (95% CI: 0–27) (Fig 3). High level of heterogeneity was observed (I2 > 90%) and this could not be reduced through subgroup analysis by sub-region nor schistosome species. No significant publication bias was observed both from the funnel plot and doi plot as shown by the LFK index of 1.83 which indicates minor asymmetry (S1 and S2 Figs).

## Discussion

Our findings reinforce observations that have been made in several epidemiological surveys regarding the transmission of schistosomiasis among PreSAC. This contributes to the growing evidence that schistosomiasis infection in high endemicity settings cuts across all age groups, thus justifying the need to include PreSAC in disease monitoring and control programmes [18, 59, 70, 71]. Considering the operational difficulties associated with collection of samples for parasitology from PreSAC [18, 72], the prevalence of schistosomiasis among PreSAC may even be higher, a potential reflection of their continued exclusion from MDA programmes [17, 73]. Although studies examining the prevalence of schistosomiasis have been reporting infection among several other age groups for several years, global interests in determining and controlling infection among PreSAC has surged lately. Our study findings support the current global agenda of improving child health and advocates for the inclusion of PreSAC in regional and national mass drug administration with praziquantel for schistosomiasis control [15, 71].

We observed that the prevalence estimates for *S*. *mansoni* (IVhet PPE: 22% (95% CI: 9–36) were higher than for *S*. *haematobium* (IVhet PPE: 15%, 95% CI: 6–25). Contrary to our observations, an earlier study that used a Bayesian geostatistical modelling approach reported prevalence levels of 17·4% and 8% for *S haematobium* and *S mansoni*, respectively among SAC [74]. According to these authors, this reduction could have resulted from MDA programmes that had been on-going and focusing on SAC [10, 11]. We think that the prevalence estimates we obtained may be explained by the reduced geographic coverage for MDA campaigns and difficulties associated with accessing praziquantel in certain endemic settings. Also, the design of MDA campaigns has focused on SAC excluding other age groups such as PreSAC [17]. Furthermore, our results are based on specific study sites survey data which may not be representative of the entire countries where the studies were done. Our results further show that the risk of infection in Central and Southern Africa are relatively lower compared to Eastern and Western Africa. The paucity of studies focusing on schistosomiasis among PreSAC in Central Africa may have led to the low estimated PPE. No studies focusing on PreSAC were obtained from countries like Botswana, Lesotho, Namibia, and Zambia in Southern Africa, thus reinforcing the fact that PreSAC are generally excluded from schistosomiasis control initiatives.

The high prevalence estimates we observed may also be due to delayed detection of infection among PreSAC despite evidence that infections can occur in children as young as 6 months old [59]. A study conducted by Albonico et al. [75] suggested that many PreSAC may carry infection for several years until they reach the school-going age and become enrolled in school. This is because disease monitoring and surveillance are more intense in the SAC group [12, 13]. Furthermore, disease control activities are often integrated into school-health programmes that take advantage of existing infrastructure in form of schools for cost-effectiveness in implementation of activities [14]. An extensive review conducted by Osakunor et al. [72] argued that schistosomiasis infections may perpetuate and go undetected or unnoticed in PreSAC due to operational difficulties and insufficient knowledge about the predisposing factors of infection in this age group. Also, Sacolo-Gwebu et al. [76], Mwai et al. [77] and Anguza et al. [78] found that poor knowledge, attitudes and misconceptions about schistosomiasis among caregivers increased the risks of infection among PreSAC. In addition, Mduluza and Mutapi [18] suggested that clinical misdiagnosis of infection owing to similarities in the clinical manifestation between schistosomiasis and other infections may explain the high disease prevalence in PreSAC.

We caution that the prevalence estimates we have reported in this paper be interpreted as indicators and not absolute values. This is because most of the studies used the Kato-Katz technique and filtration methods for the detection of infection. Detection of infection in many poor settings has heavily relied on the Kato-Katz technique for *S*. *mansoni* and *S*. *japonicum* [79, 80] and filtration methods for *S*. *haematobium* [81]. These methods have low sensitivity, thus affecting the detection of infection in children whose infection intensity may be low [79, 80, 82, 83] and that tends to under-estimate positive cases [84, 85]. The development of new infection diagnostic tools such as urine-based POC-CCA cassette test, FLOTAC technique and serological detection based on anti-schistosome antibodies [81] may increase diagnostic accuracy even among PreSAC [37, 86, 87]. Furthermore, Sousa-Figueiredo et al. [68] observed that poverty and underfunding of health systems characterise communities where schistosomiasis infection among PreSAC thrives. Lack of basic diagnostic equipment for infection diagnosis has also been observed despite having competent health workers [73]. Exclusion of PreSAC in treatment campaigns may result in growth retardation and impaired cognitive development for some of the children [5, 6, 88], and other complications like prostate cancer that manifest in adulthood [18, 89].

If schistosomiasis control programmes are to achieve the goal of disease elimination [12, 90], there is a need to expand geographical coverage and improve the delivery of chemotherapy to all age groups. Various efficacy studies [70, 91, 92] have shown the safety and benefits of praziquantel in PreSAC. Furthermore, there is a need to encourage member countries to adopt the WHO recommendation of treating PreSAC [93] once the paediatric formulation of praziquantel which is under development becomes available. Therefore, to achieve total coverage of this group in MDA and schistosomiasis control activities, establishing the prevalence of infection and risks of infection and its associated factors is essential. In addition, the collaboration of various programmes implementing preventive chemotherapy at different scales and to the same/different players and scaling up of these activities will be essential in meeting the set targets and improving perceptions about schistosomiasis [9]. The levels of infection observed in the current study indicates the need to go beyond preventive measures to integrated control that also includes health education, provision of clean water and improvement of sanitation and snail control.

## Conclusion

The results obtained in our study are of clinical and public health importance as they confirm schistosome infection among PreSAC and highlight the need for anthelminthic treatment for this age group. If countries are to achieve the goal of eliminating schistosomiasis as a public health problem as set out in the NTD road map, there is a need to include PreSAC in schistosomiasis treatment programmes.

## Supporting information

S1 File Full electronic boolean search strategy used to identify studies with all search terms and limits for at least one database and the dates on which the database was accessed to obtain the data. (DOCX)

S2 File A quality assessment tool. (DOCX)

S1 Fig Doi plot the double arcsine transformed prevalence estimate of schistosomiasis among PreSAC in SSA (LFK index: 1.89). (TIF)

S2 Fig Funnel plot of the double arcsine transformed prevalence estimates of schistosomiasis among PreSAC in SSA. (TIF)

S1 Data (XLSX)

S1 Checklist PRISMA 2009 checklist. (DOC)

## References

[1] Mackey TK, Liang BA, Cuomo R, Hafen R, Brouwer KC, Lee DE. Emerging and reemerging neglected tropical diseases: a review of key characteristics, risk factors, and the policy and innovation environment. Clinical microbiology reviews. 2014;27(4):949–79. 10.1128/CMR.00045-14 25278579 PMC4187634

[2] Steinmann P, Keiser J, Bos R, Tanner M, Utzinger J. Schistosomiasis and water resources development: systematic review, meta-analysis, and estimates of people at risk. The Lancet Infectious Diseases. 2006;6(7):411–25. 10.1016/S1473-3099(06)70521-7 16790382

[3] Lenk EJ, Redekop WK, Luyendijk M, Rijnsburger AJ, Severens JL. Productivity loss related to neglected tropical diseases eligible for preventive chemotherapy: a systematic literature review. PLoS neglected tropical diseases. 2016;10(2). 10.1371/journal.pntd.0004397 26890487 PMC4758606

[4] Simon G. Impacts of neglected tropical disease on incidence and progression of HIV/AIDS, tuberculosis, and malaria: scientific links. International Journal of Infectious Diseases. 2016;42:54–7. 10.1016/j.ijid.2015.11.006 26594012

[5] Ezeamama A, Bustinduy A, Nkwata A, Martinez L, Pabalan N, King C. Cognitive deficits and loss of education with human Schistosoma species infection–a systematic review and meta-analysis. PLoS Negl Trop Dis. 2017. 10.1371/journal.pntd.0005524 PMC5766129 29329293

[6] Ezeamama AE, Friedman JF, Acosta LP, Bellinger DC, Langdon GC, Manalo DL, et al Helminth infection and cognitive impairment among Filipino children. The American journal of tropical medicine and hygiene. 2005;72(5):540–8. 15891127 PMC1382476

[7] WHO. World health statistics 2016: monitoring health for the SDGs sustainable development goals: World Health Organization; 2016.

[8] Howden-Chapman P, Siri J, Chisholm E, Chapman R, Doll CN, Capon A. SDG 3: Ensure healthy lives and promote wellbeing for all at all ages. A guide to SDG interactions: from science to implementation Paris, France: International Council for Science. 2017:81–126.

[9] Savioli L, Albonico M, Colley DG, Correa-Oliveira R, Fenwick A, Green W, et al Building a global schistosomiasis alliance: an opportunity to join forces to fight inequality and rural poverty. Infectious Diseases of Poverty. 2017;6(1):65 10.1186/s40249-017-0280-8 28330495 PMC5363045

[10] Fenwick A, Webster JP, Bosque-Oliva E, Blair L, Fleming F, Zhang Y, et al The Schistosomiasis Control Initiative (SCI): rationale, development and implementation from 2002–2008. Parasitology. 2009;136(13):1719–30. 10.1017/S0031182009990400 19631008

[11] Savioli L, Gabrielli A, Montresor A, Chitsulo L, Engels D. Schistosomiasis control in Africa: 8 years after World Health Assembly Resolution 54.19. Parasitology. 2009;136:1677–81. 10.1017/S0031182009991181 19765347 PMC5642868

[12] WHO. Schistosomiasis: progress report 2001–2011 strategic plan 2012–2020. Geneva: World Health Organization, 2013 9241503173.

[13] WHO. Prevention and control of schistosomiasis and soil-transmitted helminthiasis: WHO Technical Report Geneva: World Health Organization, 2002 Contract No.: 912. 12592987

[14] Bundy D, Guyatt H. Schools for health: focus on health, education and the school-age child. Parasitol Today. 1996;12:1–16. 10.1016/0169-4758(96)30011-2 15275179

[15] Olsen A. Experience with school-based interventions against soil-transmitted helminths and extension of coverage to non-enrolled children. Acta Tropica. 2003;86(2–3):255–66. 10.1016/s0001-706x(03)00046-9 12745142

[16] Talaat M, Omar M, Evans D. Developing strategies to control schistosomiasis morbidity in nonenrolled school‐age children: experience from Egypt. Tropical Medicine & International Health. 1999;4(8):551–6. 10.1046/j.1365-3156.1999.00439.x 10499078

[17] Faust CL, Osakunor DN, Downs JA, Kayuni S, Stothard JR, Lamberton PH, et al Schistosomiasis Control: Leave No Age Group Behind. Trends in Parasitology. 2020 10.1016/j.pt.2020.04.012 32430274 PMC7905337

[18] Mduluza T, Mutapi F. Putting the treatment of paediatric schistosomiasis into context. Infectious diseases of poverty. 2017;6(1):85 10.1186/s40249-017-0300-8 28388940 PMC5384153

[19] Moher D, Liberati A, Tetzlaff J, Altman DG. Preferred reporting items for systematic reviews and meta-analyses: the PRISMA statement. Annals of internal medicine. 2009;151(4):264–9. 10.7326/0003-4819-151-4-200908180-00135 19622511

[20] Munn Z, Moola S, Riitano D, Lisy K. The development of a critical appraisal tool for use in systematic reviews addressing questions of prevalence. Int J Health Policy Manag. 2014;3(3):123–8. 10.15171/ijhpm.2014.71 25197676 PMC4154549

[21] Doi S, Barendregt J, Khan S, Thalib L, Williams G. Advances in the Meta-analysis of heterogeneous clinical trials I: The inverse variance heterogeneity model. Contemp Clin Trials. 2015;45:130–8. 10.1016/j.cct.2015.05.009 26003435

[22] Barendregt J, Doi S. MetaXL User Guide version 5.3. EpiGear International Pty Ltd. 2016.

[23] Macklina G, Michelle CS, Albert T-T, Russell S. A pilot study using wearable global positioning system data loggers to compare water contact levels: Schistosoma haematobium infection in pre-school-age children (PSAC) and their mothers at Barombi Kotto, Cameroon. Trans R Soc Trop Med Hyg 2018;112:361–5. 10.1093/trstmh/try059 29992295

[24] Moyo V, Changadeya W, Chiotha S, Sikawa D. Urinary schistosomiasis among preschool children in Malengachanzi, Nkhotakota District, Malawi: prevalence and risk factors. Malawi Medical Journal. 2016;28(1):10–4. 10.4314/mmj.v28i1.3 27217911 PMC4864386

[25] Poole H, Terlouw DJ, Naunje A, Mzembe K, Stanton M, Betson M, et al Schistosomiasis in pre-school-age children and their mothers in Chikhwawa district, Malawi with notes on characterization of schistosomes and snails. Parasites & vectors. 2014;7(1):153 10.1186/1756-3305-7-153 24690282 PMC4230191

[26] Wami WM, Nausch N, Midzi N, Gwisai R, Mduluza T, Woolhouse M, et al Identifying and evaluating field indicators of urogenital schistosomiasis-related morbidity in preschool-aged children. PLoS neglected tropical diseases. 2015;9(3). 10.1371/journal.pntd.0003649 25793584 PMC4368198

[27] Osakunor DNM, Mduluza T, Midzi N, Chase-Topping M, Mutsaka-Makuvaza MJ, Chimponda T, et al Dynamics of paediatric urogenital schistosome infection, morbidity and treatment: a longitudinal study among preschool children in Zimbabwe. BMJ global health. 2018;3(2):e000661 10.1136/bmjgh-2017-000661 29616147 PMC5875666

[28] Chu T, Liao C, D’Lamini P, Chang P, Chiu W, Du W, et al Prevalence of Schistosoma haematobium infection among inhabitants of Lowveld, Swaziland, an endemic area for the disease. Tropical biomedicine. 2010;27(2):337–42. 20962734

[29] Sacolo-Gwebu H, Moses C, Chester K. Prevalence and risk factors of schistosomiasis and soil-transmitted helminthiases among preschool aged children (1–5 years) in rural KwaZulu-Natal, South Africa: a cross-sectional study. Infect Dis Poverty. 2019 10.1186/s40249-019-0561-5 31202273 PMC6571117

[30] Mutsaka-Makuvaza MJ, Zvifadzo M-Z, Cremance T, Sunanda R, Xiao-Nong Z, Bonnie W, et al Reinfection of urogenital schistosomiasis in pre-school children in a highly endemic district in Northern Zimbabwe: a 12 months compliance study. Infect Dis Poverty. 2018;7:102 10.1186/s40249-018-0483-7 30268157 PMC6162945

[31] Sousa-Figueiredo JC, Gamboa D, Pedro JM, Fançony C, Langa AJ, Magalhães RJS, et al Epidemiology of malaria, schistosomiasis, geohelminths, anemia and malnutrition in the context of a demographic surveillance system in northern Angola. PLoS One. 2012;7(4). 10.1371/journal.pone.0033189 22493664 PMC3320883

[32] Chimponda TN, Mduluza T. Inflammation during Schistosoma haematobium infection and anti‐allergy in pre‐school‐aged children living in a rural endemic area in Zimbabwe. Tropical Medicine & International Health. 2020;25(5):618–23. 31990094 10.1111/tmi.13376

[33] Mduluza-Jokonya TL, Naicker T, Jokonya L, Midzi H, Vengesai A, Kasambala M, et al Association of Schistosoma haematobium infection morbidity and severity on co-infections in pre-school age children living in a rural endemic area in Zimbabwe. BMC public health. 2020;20(1):1–9. 33076903 10.1186/s12889-020-09634-0 PMC7574170

[34] Adeniran AA, Mogaji HO, Aladesida AA, Olayiwola IO, Oluwole AS, Abe EM, et al Schistosomiasis, intestinal helminthiasis and nutritional status among preschool-aged children in sub-urban communities of Abeokuta, Southwest, Nigeria. BMC research notes. 2017;10(1):637 10.1186/s13104-017-2973-2 29183397 PMC5706406

[35] Babatunde T, Asaolu S, Sowemimo O. Urinary schistosomiasis among pre-school and school aged children in two peri-urban communities in Southwest Nigeria. Journal of parasitology and vector biology. 2013;5(7):96–101.

[36] Bosompem K, Bentum IA, Otchere J, Anyan W, Brown C, Osada Y, et al Infant schistosomiasis in Ghana: a survey in an irrigation community. Tropical medicine & international health. 2004;9(8):917–22. 15303998 10.1111/j.1365-3156.2004.01282.x

[37] Coulibaly JT, N’gbesso YK, Knopp S, N’guessan NA, Silué KD, van Dam GJ, et al Accuracy of urine circulating cathodic antigen test for the diagnosis of Schistosoma mansoni in preschool-aged children before and after treatment. PLoS neglected tropical diseases. 2013;7(3). 10.1371/journal.pntd.0002109 PMC3605147 23556011

[38] Dabo A, Badawi HM, Bary B, Doumbo OK. Urinary schistosomiasis among preschool-aged children in Sahelian rural communities in Mali. Parasites & vectors. 2011;4(1):21 10.1186/1756-3305-4-21 21338486 PMC3058107

[39] Ekpo UF, Laja-Deile A, Oluwole AS, Sam-Wobo SO, Mafiana CF. Urinary schistosomiasis among preschool children in a rural community near Abeokuta, Nigeria. Parasites & vectors. 2010;3(1):58. 20602792 10.1186/1756-3305-3-58 PMC2908585

[40] Garba A, Barkiré N, Djibo A, Lamine MS, Sofo B, Gouvras AN, et al Schistosomiasis in infants and preschool-aged children: infection in a single Schistosoma haematobium and a mixed S. haematobium–S. mansoni foci of Niger. Acta tropica. 2010;115(3):212–9. 10.1016/j.actatropica.2010.03.005 20303925

[41] Hodges MH, Paye J, Koroma MM, Nyorkor ED, Fofonah I, Zhang Y. High level of Schistosoma mansoni infection in pre-school children in Sierra Leone highlights the need in targeting this age group for praziquantel treatment. Acta tropica. 2012;124(2):120–5. 10.1016/j.actatropica.2012.07.005 22820025

[42] Houmsou R, Agere H, Wama B, Bingbeng J, Amuta E, Kela S. Urinary schistosomiasis among children in Murbai and Surbai communities of Ardo-Kola local government area, Taraba state, Nigeria. Journal of tropical medicine. 2016;2016 10.1155/2016/9831265 28096819 PMC5206853

[43] Mafiana C, Ekpo U, Ojo D. Urinary schistosomiasis in preschool children in settlements around Oyan Reservoir in Ogun State, Nigeria: implications for control. Tropical Medicine & International Health. 2003;8(1):78–82. 10.1046/j.1365-3156.2003.00988.x 12535255

[44] Opara K, Udoidung N, Ukpong I. Genitourinary schistosomiasis among pre-primary schoolchildren in a rural community within the Cross River Basin, Nigeria. Journal of Helminthology. 2007;81(4):393–7. 10.1017/S0022149X07853521 18005467

[45] Salawu TO, Alexander BO. Urogenital schistosomiasis and urological assessment of hematuria in preschool-aged children in rural communities of Nigeria. Journal of Pediatric Urology. 2013;XX:1–6. 10.1016/j.jpurol.2013.06.010 23891456

[46] Armoo S, Cunningham LJ, Campbell SJ, Aboagye FT, Boampong FK, Hamidu BA, et al Detecting Schistosoma mansoni infections among pre-school-aged children in southern Ghana: a diagnostic comparison of urine-CCA, real-time PCR and Kato-Katz assays. BMC Infectious Diseases. 2020;20:1–10. 10.1186/s12879-020-05034-2 32321418 PMC7178570

[47] Ekpo U, Alabi O, Oluwole A, Sam-Wobo S. Schistosoma haematobium infections in preschool children from two rural communities in Ijebu East, south-western Nigeria. Journal of helminthology. 2012;86(3):323–8. 10.1017/S0022149X11000459 22824258

[48] Ekpo U, Fafunwa T, Oluwole A, Abe E, Mafiana C. Prevalence and factors associated with urinary schistosomiasis among infants and preschool-aged children in settlements around Oyan reservoir in Ogun State, Nigeria. Journal of Natural Sciences, Engineering, and Technology. 2012;11:82–92.

[49] Alemu A, Tegegne Y, Damte D, Melku M. Schistosoma mansoni and soil-transmitted helminths among preschool-aged children in Chuahit, Dembia district, Northwest Ethiopia: prevalence, intensity of infection and associated risk factors. BMC Public Health. 2016;16(1):422. 27216255 10.1186/s12889-016-2864-9 PMC4876558

[50] Alemu M, Bedemo H, Bugssa G, Bayissa S, Tedla K. Epidemiology of intestinal parasite infections among kindergarten children in Mekelle Town, Northern Ethiopia. Int J Pharma Sci Res. 2015;6(11):1392–6.

[51] Betson M, Sousa-Figueiredo JC, Rowell C, Kabatereine NB, Stothard JR. Intestinal schistosomiasis in mothers and young children in Uganda: investigation of field-applicable markers of bowel morbidity. The American journal of tropical medicine and hygiene. 2010;83(5):1048–55. 10.4269/ajtmh.2010.10-0307 21036836 PMC2963968

[52] Mueller A, Fuss A, Ziegler U, Kaatano GM, Mazigo HD. Intestinal schistosomiasis of Ijinga Island, north-western Tanzania: prevalence, intensity of infection, hepatosplenic morbidities and their associated factors. BMC infectious diseases. 2019;19(1):832 10.1186/s12879-019-4451-z 31590657 PMC6781372

[53] Nalugwa A, Nuwaha F, Tukahebwa EM, Olsen A. Schistosoma mansoni-Associated morbidity among preschool-aged children along the shores of Lake Victoria in Uganda. Tropical medicine and infectious disease. 2017;2(4):58. 10.3390/tropicalmed2040058 PMC6082064 30270915

[54] Ndokeji SD, Mazigo HD, Temu M, Kishamawe C, Malenganisho W, Todd J, et al Prevalence and intensity of Schistosoma mansoni and hookworm infections among pre-school and school-aged children in Ilemela District, north-western Tanzania. Tanzania Journal of Health Research. 2016;18(2).

[55] Niyituma E, Njunwa KJ, Mbugua AK, Karema C, Umulisa I, Mkoji GM. Intestinal schistosomiasis and the associated transmission factors in pre-school aged children in villages surrounding Lake Rweru in Bugesera District, Rwanda. Rwanda Journal. 2017;4(1):29–35.

[56] Pinot de Moira A, Sousa-Figueiredo JC, Jones FM, Fitzsimmons CM, Betson M, Kabatereine NB, et al Schistosoma mansoni infection in preschool-aged children: development of immunoglobulin E and immunoglobulin G4 responses to parasite allergen-like proteins. The Journal of infectious diseases. 2013;207(2):362–6. 10.1093/infdis/jis676 23125445 PMC3532835

[57] Ruganuza DM, Mazigo HD, Waihenya R, Morona D, Mkoji GM. Schistosoma mansoni among pre-school children in Musozi village, Ukerewe Island, North-Western-Tanzania: prevalence and associated risk factors. Parasites & vectors. 2015;8(1):377 10.1186/s13071-015-0997-9 26178484 PMC4504164

[58] Stothard JR, Sousa-Figueiredo JC, Betson M, Seto EY, Kabatereine NB. Investigating the spatial micro-epidemiology of diseases within a point-prevalence sample: a field applicable method for rapid mapping of households using low-cost GPS-dataloggers. Transactions of the Royal Society of Tropical Medicine and Hygiene. 2011;105(9):500–6. 10.1016/j.trstmh.2011.05.007 21714979 PMC3183225

[59] Stothard JR, Sousa-Figuereido JC, Betson M, Adriko M, Arinaitwe M, Rowell C, et al Schistosoma mansoni infections in young children: when are schistosome antigens in urine, eggs in stool and antibodies to eggs first detectable? PLoS neglected tropical diseases. 2011;5(1):e938 10.1371/journal.pntd.0000938 21245910 PMC3014943

[60] Verani JR, Abudho B, Montgomery SP, Mwinzi PN, Shane HL, Butler SE, et al Schistosomiasis among young children in Usoma, Kenya. The American journal of tropical medicine and hygiene. 2011;84(5):787–91. 10.4269/ajtmh.2011.10-0685 21540390 PMC3083748

[61] Sakari SS, Amos KM, Gerald MM. Prevalence of Soil-Transmitted Helminthiases and Schistosomiasis in Preschool Age Children in Mwea Division, Kirinyaga South District, Kirinyaga County, and Their Potential Effect on Physical Growth. Journal of Tropical Medicine. 2017 10.1155/2017/1013802 29138640 PMC5613645

[62] Kemal M, Gemechu T, Adem E, Solomon MA, Tadesse K. Schistosoma mansoni infection among preschool age children attending Erer Health Center, Ethiopia and the response rate to praziquantel. BMC Research Notes. 2019;12:211 10.1186/s13104-019-4246-8 30953565 PMC6451229

[63] Odogwu S, Ramamurthy N, Kabatereine N, Kazibwe F, Tukahebwa E, Webster J, et al Schistosoma mansoni in infants (aged, 3 years) along the Ugandan shoreline of Lake Victoria. Annals of Tropical Medicine & Parasitology. 2006;100(4). 10.1179/136485906X105552 16762112

[64] Rujeni N, Alex M, Musafiri T, Elias N, Gad R, Pascal K, et al Pre-school aged children are exposed to Schistosoma through Lake Kivu in Rwanda. AAS Open Research. 2019;2(7):1–13. 10.12688/aasopenres.12930.1 36419723 PMC9648362

[65] G/hiwot Y, Abraham D, Berhanu E. Prevalence of Intestinal Parasitic Infections among Children under Five Years of Age with Emphasis on Schistosoma mansoni in Wonji Shoa Sugar Estate, Ethiopia. PLoS ONE. 2014;(10):e109793 10.1371/journal.pone.0109793 25296337 PMC4190315

[66] Masaku J, Njomo DW, Njoka A, Okoyo C, Mutungi FM, Njenga SM. Soil-transmitted helminths and schistosomiasis among pre-school age children in a rural setting of Busia County, Western Kenya: a cross-sectional study of prevalence, and associated exposures. BMC public health. 2020;20(1):1–11. 32188444 10.1186/s12889-020-08485-z PMC7079432

[67] Sassa M, Chadeka EA, Cheruiyot NB, Tanaka M, Moriyasu T, Kaneko S, et al Prevalence and risk factors of Schistosoma mansoni infection among children under two years of age in Mbita, Western Kenya. PLoS neglected tropical diseases. 2020;14(8):e0008473 10.1371/journal.pntd.0008473 32841228 PMC7447014

[68] Sousa-Figueiredo JC, Pleasant J, Day M, Betson M, Rollinson D, Montresor A, et al Treatment of intestinal schistosomiasis in Ugandan preschool children: best diagnosis, treatment efficacy and side-effects, and an extended praziquantel dosing pole. International Health. 2010;2(2):103–13. 10.1016/j.inhe.2010.02.003 20640034 PMC2892744

[69] Lewetegn M, Getachew M, Kebede T, Tadesse G, Asfaw T. Prevalence of Intestinal Parasites Among Preschool Children and Maternal KAP on Prevention and Control in Senbete and Bete Towns, North Shoa, Ethiopia. Int J Biomed Mater Res. 2019;7(1):1–7.

[70] Sousa-Figueiredo JC, Betson M, Stothard JR. Treatment of schistosomiasis in African infants and preschool-aged children: downward extension and biometric optimization of the current praziquantel dose pole. International health. 2012;4(2):95–102. 10.1016/j.inhe.2012.03.003 22876272 PMC3407873

[71] Mutapi F. Changing policy and practice in the control of pediatric schistosomiasis. Pediatrics. 2015:peds. 2014-3189. 10.1542/peds.2014-3189 25687146

[72] Osakunor DN, Woolhouse ME, Mutapi F. Paediatric schistosomiasis: What we know and what we need to know. PLoS neglected tropical diseases. 2018;12(2):e0006144 10.1371/journal.pntd.0006144 29420537 PMC5805162

[73] Stothard JR, Sousa-Figueiredo JC, Betson M, Bustinduy A, Reinhard-Rupp J. Schistosomiasis in African infants and preschool children: let them now be treated! Trends in parasitology. 2013;29(4):197–205. 10.1016/j.pt.2013.02.001 23465781 PMC3878762

[74] Lai Y-S, Biedermann P, Ekpo UF, Garba A, Mathieu E, Midzi N, et al Spatial distribution of schistosomiasis and treatment needs in sub-Saharan Africa: a systematic review and geostatistical analysis. The Lancet infectious diseases. 2015;15(8):927–40. 10.1016/S1473-3099(15)00066-3 26004859

[75] Albonico M, Crompton D, Savioli L. Control strategies for human intestinal nematode infections. Advances in Parasitology. 1999;42:277–431. 10.1016/s0065-308x(08)60151-7 10050275

[76] Sacolo-Gwebu H, Kabuyaya M, Chimbari M. Knowledge, attitudes and practices on schistosomiasis and soil-transmitted helminths among caregivers in Ingwavuma area in uMkhanyakude district, South Africa. BMC infectious diseases. 2019;19(1):734 10.1186/s12879-019-4253-3 31438865 PMC6704662

[77] Mwai J, Njenga S, Barasa M. Knowledge, attitude and practices in relation to prevention and control of schistosomiasis infection in Mwea Kirinyaga county, Kenya. BMC public health. 2016;16(1):819 10.1186/s12889-016-3494-y 27538885 PMC4991016

[78] Anguza J, Oryema-Lalobo M, Oundo G, Nuwaha F. Community perception of intestinal schistosomiasis in Busia district of Uganda. East African medical journal. 2007;84(2):56–66. 10.4314/eamj.v84i2.9505 17598666

[79] Knopp S, Speich B, Hattendorf J, Rinaldi L, Mohammed KA, Khamis IS, et al Diagnostic accuracy of Kato-Katz and FLOTAC for assessing anthelmintic drug efficacy. PLoS neglected tropical diseases. 2011;5(4). 10.1371/journal.pntd.0001036 21532740 PMC3075226

[80] Lin D-D, Liu J-X, Liu Y-M, Hu F, Zhang Y-Y, Xu J-M, et al Routine Kato–Katz technique underestimates the prevalence of Schistosoma japonicum: a case study in an endemic area of the People’s Republic of China. Parasitology international. 2008;57(3):281–6. 10.1016/j.parint.2008.04.005 18485807

[81] Utzinger J, Becker S, Van Lieshout L, Van Dam G, Knopp S. New diagnostic tools in schistosomiasis. Clinical microbiology and infection. 2015;21(6):529–42. 10.1016/j.cmi.2015.03.014 25843503

[82] Braun-Munzinger R, Southgate B. Repeatability and reproducibility of egg counts of Schistosoma haematobium in urine. Tropical medicine and parasitology: official organ of Deutsche Tropenmedizinische Gesellschaft and of Deutsche Gesellschaft fur Technische Zusammenarbeit (GTZ). 1992;43(3):149–54. 1470831

[83] Melchers NVV, van Dam GJ, Shaproski D, Kahama AI, Brienen EA, Vennervald BJ, et al Diagnostic performance of Schistosoma real-time PCR in urine samples from Kenyan children infected with Schistosoma haematobium: day-to-day variation and follow-up after praziquantel treatment. PLoS neglected tropical diseases. 2014;8(4). 10.1371/journal.pntd.0002807 PMC3990496 24743389

[84] Bergquist R, Johansen MV, Utzinger J. Diagnostic dilemmas in helminthology: what tools to use and when? Trends in parasitology. 2009;25(4):151–6. 10.1016/j.pt.2009.01.004 19269899

[85] Van Lieshout L, Polderman A, Deelder A. Immunodiagnosis of schistosomiasis by determination of the circulating antigens CAA and CCA, in particular in individuals with recent or light infections. Acta tropica. 2000;77(1):69–80. 10.1016/s0001-706x(00)00115-7 10996122

[86] Colley DG, Binder S, Campbell C, King CH, Tchuenté L-AT, N’Goran EK, et al A five-country evaluation of a point-of-care circulating cathodic antigen urine assay for the prevalence of Schistosoma mansoni. The American journal of tropical medicine and hygiene. 2013;88(3):426–32. 10.4269/ajtmh.12-0639 23339198 PMC3592520

[87] Kittur N, Castleman JD, Campbell CH Jr, King CH, Colley DG. Comparison of Schistosoma mansoni prevalence and intensity of infection, as determined by the circulating cathodic antigen urine assay or by the Kato-Katz fecal assay: a systematic review. The American journal of tropical medicine and hygiene. 2016;94(3):605–10. 10.4269/ajtmh.15-0725 26755565 PMC4775897

[88] King CH, Dangerfield-Cha M. The unacknowledged impact of chronic schistosomiasis. Chronic illness. 2008;4(1):65–79. 10.1177/1742395307084407 18322031

[89] Barda B, Coulibaly JT, Hatz C, Keiser J. Ultrasonographic evaluation of urinary tract morbidity in school-aged and preschool-aged children infected with Schistosoma haematobium and its evolution after praziquantel treatment: A randomized controlled trial. PLoS neglected tropical diseases. 2017;11(2):e0005400 10.1371/journal.pntd.0005400 28222149 PMC5336295

[90] Tchuem Tchuenté L, Rollinson D, Stothard J, Molyneux D. Moving from control to elimination of schistosomiasis in sub-Saharan Africa: time to change and adapt strategies. Infect Dis Poverty. 2017;6(1):42 10.1186/s40249-017-0256-8 28219412 PMC5319063

[91] Mutapi F, Rujeni N, Bourke C, Mitchell K, Appleby L, Nausch N, et al Schistosoma haematobium treatment in 1–5 year old children: safety and efficacy of the antihelminthic drug praziquantel. PLoS neglected tropical diseases. 2011;5(5). 10.1371/journal.pntd.0001143 21610855 PMC3096601

[92] Wami WM, Norman N, Nicholas M, Reggis G, Takafira M, Mark W, et al Comparative Assessment of Health Benefits of Praziquantel Treatment of Urogenital Schistosomiasis in Preschool and Primary School-Aged Children. BioMed Research International. 2016:1–11. 10.1155/2016/9162631 27631011 PMC5007301

[93] WHO. Report of a meeting to review the results of studies on the treatment of schistosomiasis in preschool-age children. Geneva: World Health Organization, 2011 924150188X.

