# Supplementary material for: A systematic review and meta-analysis quantifying schistosomiasis infection burden in pre-school aged children (PreSAC) in sub-Saharan Africa for the period 2000–2020
Source: PLoS One. 2020 Dec 29;15(12):e0244695. doi: 10.1371/journal.pone.0244695 (PMC7771669; doi:10.1371/journal.pone.0244695)
Supplement: S1 File — (DOCX) [file pone.0244695.s001.docx]

**Full electronic Boolean search strategy used to identify studies with all search terms and limits for at least one database and the dates on which the database was accessed to obtain the data**

**Google scholar (30^th^ November 2020)**

Google scholar Search phrase: schistosomiasis among pre-school aged children in sub-Saharan Africa (up to 10 pages)

Google scholar search phrase for: “schistosomiasis AND pre-school" OR "pre-school" OR "under five" AND "sub-Saharan Africa" NOT "school-age children" (up to page 80)

**PubMed (30^th^ November 2020)**

PubMed Search: schistosomiasis among pre-school aged children in sub-Saharan Africa

(("schistosomiasis"[MeSH Terms] OR "schistosomiasis"[All Fields]) OR "schistosomiases"[All Fields]) AND (((("child, preschool"[MeSH Terms] OR ("child"[All Fields] AND "preschool"[All Fields])) OR "preschool child"[All Fields]) OR ("pre"[All Fields] AND "school"[All Fields])) OR "pre school"[All Fields]) AND ("aged"[MeSH Terms] OR "aged"[All Fields]) AND (((((("child"[MeSH Terms] OR "child"[All Fields]) OR "children"[All Fields]) OR "child s"[All Fields]) OR "children s"[All Fields]) OR "childrens"[All Fields]) OR "childs"[All Fields]) AND (((("africa south of the sahara"[MeSH Terms] OR (("africa"[All Fields] AND "south"[All Fields]) AND "sahara"[All Fields])) OR "africa south of the sahara"[All Fields]) OR (("sub"[All Fields] AND "saharan"[All Fields]) AND "africa"[All Fields])) OR "sub saharan africa"[All Fields])

Translations

schistosomiasis: "schistosomiasis"[MeSH Terms] OR "schistosomiasis"[All Fields] OR "schistosomiases"[All Fields]

pre-school: "child, preschool"[MeSH Terms] OR ("child"[All Fields] AND "preschool"[All Fields]) OR "preschool child"[All Fields] OR ("pre"[All Fields] AND "school"[All Fields]) OR "pre school"[All Fields]

aged: "aged"[MeSH Terms] OR "aged"[All Fields]

children: "child"[MeSH Terms] OR "child"[All Fields] OR "children"[All Fields] OR "child's"[All Fields] OR "children's"[All Fields] OR "childrens"[All Fields] OR "childs"[All Fields]

sub-Saharan Africa: "africa south of the sahara"[MeSH Terms] OR ("africa"[All Fields] AND "south"[All Fields] AND "sahara"[All Fields]) OR "africa south of the sahara"[All Fields] OR ("sub"[All Fields] AND "saharan"[All Fields] AND "africa"[All Fields]) OR "sub saharan africa"[All Fields]
