# Supplementary material for: A systematic review and meta-analysis quantifying schistosomiasis infection burden in pre-school aged children (PreSAC) in sub-Saharan Africa for the period 2000–2020
Source: PLoS One. 2020 Dec 29;15(12):e0244695. doi: 10.1371/journal.pone.0244695 (PMC7771669; doi:10.1371/journal.pone.0244695)
Supplement: S2 File — (DOCX) [file pone.0244695.s002.docx]

**Report of the outcome of analysis of the quality of included papers.**

For assessing the quality of all studies included in the review we used the Joanna Briggs Institute Prevalence Critical Appraisal Tool: Quality assessment tool for prevalence studies. The tool assessed the following questions about the articles:

1. sample representative of the target population,
2. study participants recruited in an appropriate way,
3. sample size adequate
4. study subjects and setting described in detail
5. data analysis conducted with enough coverage of the identified sample
6. objective standard criteria used for measurement of the condition
7. condition measured reliably,
8. appropriate statistical analysis,
9. important confounding factors/ subgroups/differences identified and accounted for,
10. subpopulations identified using objective criteria.

| First Author and Year | Was the sample representative of the target population? | Were study participants recruited in an appropriate way? | Was the sample size adequate? | Were the study subjects and setting described in detail? | Is the data analysis conducted with sufficient coverage of the identified sample? | Were objective, standard criteria used for measurement of the condition? | Was the condition measured reliably? | Was there appropriate statistical analysis? | Are all important confounding factors/ subgroups/differences identified and accounted for? | Were subpopulations identified using objective criteria? | Quality score |
| --- | --- | --- | --- | --- | --- | --- | --- | --- | --- | --- | --- |
| Adeniran et al. 2017 | Yes | Yes | Yes | Yes | Yes | Yes | Yes | Yes | Yes | Yes | 9 |
| Alemu et al. 2015 | Yes | No | Yes | No | Yes | Yes | Yes | Yes | NA | Yes | 7 |
| Alemu et al. 2016 | Yes | Yes | Yes | Yes | Yes | Yes | Yes | Yes | Yes | Yes | 10 |
| Armoo et al. 2020 | Yes | Yes | Yes | Yes | Yes | Yes | Yes | Yes | N/A | Yes | 9 |
| Babatunde et al. 2013 | Yes | Yes | Yes | Yes | Yes | Yes | Yes | Yes | NA | yes | 9 |
| Betson et al. 2010 | Yes | Yes | Yes | Yes | Yes | Yes | Yes | Yes | NA | Yes | 9 |
| Bosompem et al. 2004 | Yes | No | Yes | No | yes | Yes | Yes | Yes | Yes | Yes | 8 |
| Chiponda & Mduluza 2020 | Yes | Yes | Yes | Yes | Yes | Yes | Yes | Yes | NA | Yes | 9 |
| Coulibaly et al. 2013 | Yes | Yes | Yes | Yes | Yes | Yes | Yes | Yes | NA | Yes | 9 |
| Chu et al. 2010 | Yes | Yes | Yes | Yes | Yes | Yes | Yes | Yes | NA | Yes | 9 |
| Dabo et al. 2011 | Yes | Yes | Yes | Yes | Ys | Yes | Yes | Yes | NA | yes | 9 |
| Sousa-Figueiredo et al. 2010 | Yes | Yes | Yes | Yes | Yes | Yes | Yes | Yes | NA | Yes | 9 |
| Ekpo et al. 2010 | Yes | Yes | Yes | Yes | Yes | Yes | Yes | Yes | NA | Yes | 9 |
| Ekpo et al. 2012a | Yes | Yes | Yes | Yes | Yes | Yes | Yes | Yes | NA | Yes | 9 |
| Ekpo et al. 2012b | Yes | Yes | Yes | Yes | Yes | Yes | Yes | Yes | NA | Yes | 9 |
| G/hiwot et al. 2014 | Yes | Yes | Yes | Yes | Yes | Yes | Yes | Yes | NA | Yes | 9 |
| Garba et al. 2010 | Yes | Yes | Yes | Yes | Yes | Yes | Yes | Yes | NA | Yes | 9 |
| Hodges et al. 2012 | Yes | Yes | Yes | Yes | Yes | Yes | Yes | Yes | NA | yes | 9 |
| Houmsou et al. 2016 | Yes | Yes | Yes | Yes | Yes | Yes | Yes | Yes | NA | Yes | 9 |
| Kemal et al. 2019 | Yes | Yes | Yes | Yes | Yes | Yes | Yes | Yes | NA | Yes | 9 |
| Lewetegn et al. 2020 | Yes | Yes | No | No | Yes | Yes | Yes | Yes | NA | N/A | 6 |
| Macklin et al. 2018 | Yes | Yes | No | No | Yes | Yes | Yes | Yes | NA | Yes | 7 |
| Mafiana 2003 | Yes | Yes | Yes | Yes | Yes | Yes | Yes | Yes | NA | Yes | 9 |
| Masaku et al. 2020 | Yes | Yes | Yes | Yes | Yes | Yes | Yes | Yes | NA | Yes | 9 |
| Mduluza-Jokonya et al. 2020 | Yes | Yes | Yes | Yes | Yes | Yes | Yes | Yes | NA | Yes | 9 |
| Moyo et al. 2016 | Yes | Yes | Yes | Yes | Yes | Yes | Yes | Yes | Yes | Yes | 10 |
| Mueller et al. 2019 | Yes | Yes | Yes | Yes | Yes | Yes | Yes | Yes | NA | Yes | 9 |
| Mutsaka-Makuvaza et al. 2018 | Yes | Yes | Yes | Yes | Yes | Yes | Yes | Yes | NA | Yes | 9 |
| Nalugwa et al. 2015 | Yes | Yes | Yes | No | Yes | Yes | Yes | Yes | NA | Yes | 8 |
| Ndokeji et al. 2016 | Yes | Yes | Yes | Yes | Yes | Yes | Yes | Yes | NA | Yes | 9 |
| Niyituma et al. 2017 | Yes | Yes | Yes | Yes | Yes | Yes | Yes | Yes | NA | Yes | 9 |
| Odogwu et al. 2006 | Yes | Yes | Yes | Yes | Yes | Yes | Yes | Yes |  | Yes | 9 |
| Opara et al. 2007 | Yes | Yes | Yes | Yes | Yes | Yes | Yes | Yes | NA | Yes | 9 |
| Osakunor et al. 2018 | Yes | Yes | Yes | Yes | Yes | Yes | Yes | Yes | NA | Yes | 9 |
| Pinot de Moira et al. 2012 | Yes | Yes | Yes | No | Yes | Yes | Yes | Yes | NA | Yes | 8 |
| Poole et al. 2014 | Yes | Yes | Yes | Yes | Yes | Yes | Yes | Yes | NA | Yes | 9 |
| Ruganuza et al. 2015 | Yes | Yes | Yes | Yes | Yes | Yes | Yes | Yes | NA | Yes | 9 |
| Rujeni et al. 2019 | Yes | Yes | Yes | Yes | Yes | Yes | Yes | Yes | NA | Yes | 9 |
| Sacolo-Gwebu et al. 2019 | Yes | Yes | Yes | Yes | Yes | Yes | Yes | Yes | NA | Yes | 9 |
| Sassa et al. 2020 | Yes | Yes | Yes | Yes | Yes | Yes | Yes | Yes | NA | Yes | 9 |
| Sakari et al. 2017 | Yes | Yes | Yes | Yes | Yes | Yes | Yes | Yes | NA | Yes | 9 |
| Salawu & Odaibo. 2013 | Yes | Yes | Yes | Yes | Yes | Yes | Yes | Yes | NA | Yes | 9 |
| Stothard et al. 2011a | Yes | Yes | Yes | Yes | Yes | Yes | Yes | Yes | NA | Yes | 9 |
| Stothard et al. 2011b | Yes | Yes | Yes | Yes | Yes | Yes | Yes | Yes | NA | Yes | 9 |
| Verani et al. 2011 | Yes | Yes | Yes | Yes | Yes | Yes | Yes | Yes | NA | Yes | 9 |
| Wami et al. 2015 | Yes | Yes | Yes | Yes | Yes | Yes | Yes | Yes | NA | Yes | 9 |
| Sousa-Figueiredo et al. 2012 | Yes | Yes | Yes | Yes | Yes | Yes | Yes | Yes | NA | Yes | 9 |
