# Supplementary figures and images for: A systematic review and meta-analysis quantifying schistosomiasis infection burden in pre-school aged children (PreSAC) in sub-Saharan Africa for the period 2000–2020
Source: PLoS One. 2020 Dec 29;15(12):e0244695. doi: 10.1371/journal.pone.0244695 (PMC7771669; doi:10.1371/journal.pone.0244695)

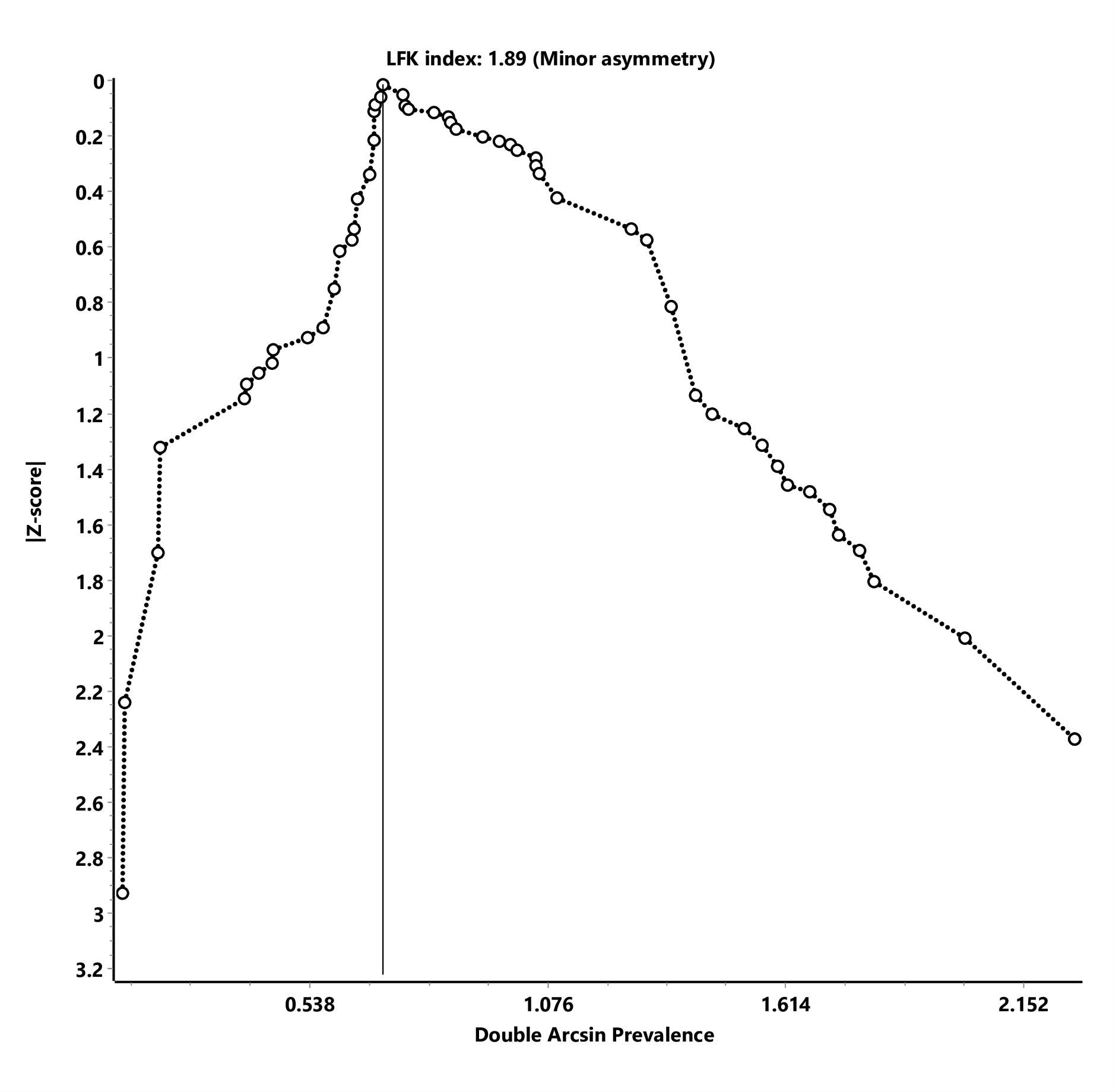

Supplement: S1 Fig — (TIF) [file pone.0244695.s003.tif]

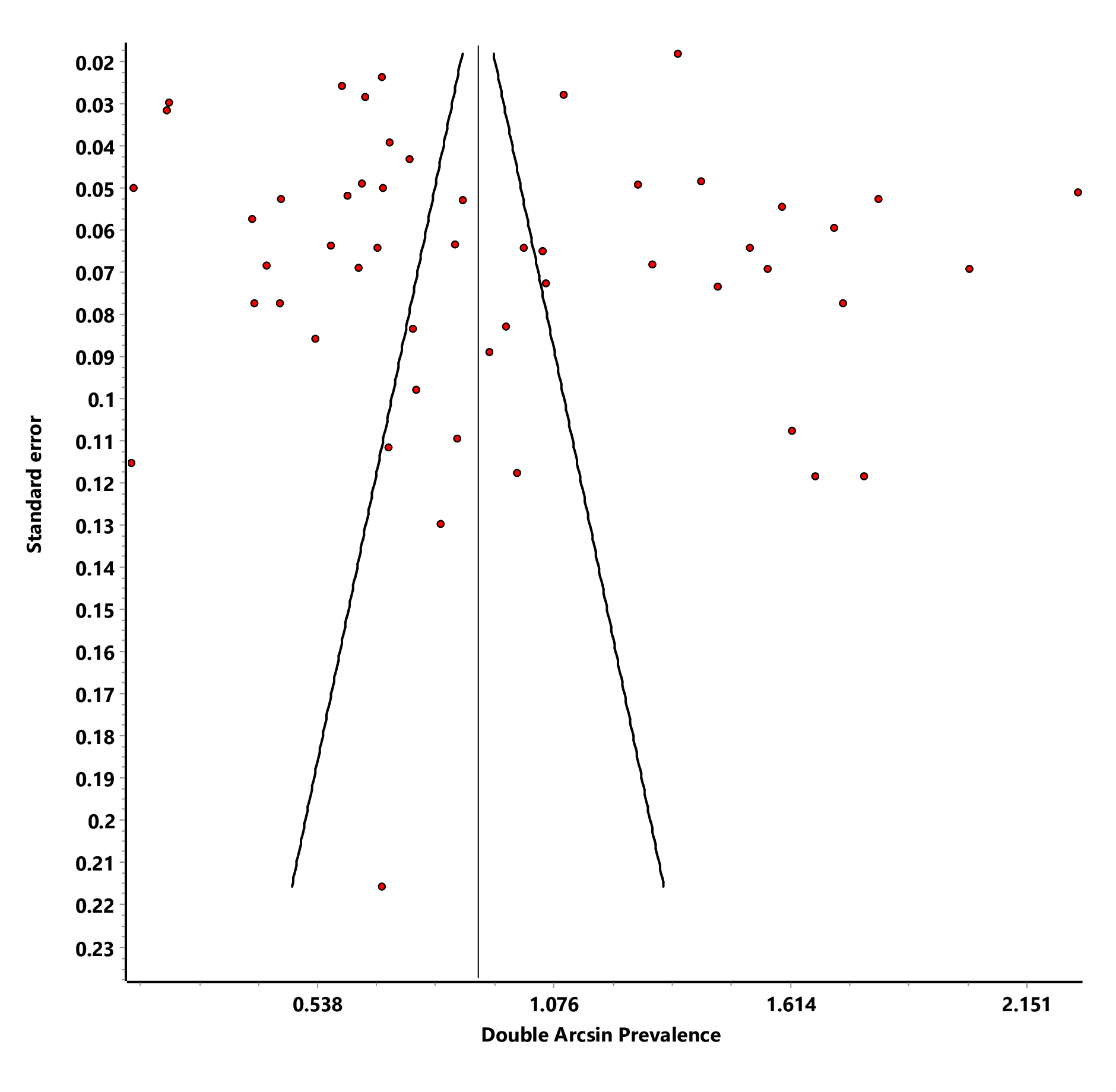

Supplement: S2 Fig — (TIF) [file pone.0244695.s004.tif]
