# Supplementary material for: A systematic review and meta-analysis quantifying schistosomiasis infection burden in pre-school aged children (PreSAC) in sub-Saharan Africa for the period 2000–2020
Source: PLoS One. 2020 Dec 29;15(12):e0244695. doi: 10.1371/journal.pone.0244695 (PMC7771669; doi:10.1371/journal.pone.0244695)
Supplement: Attachment — Submitted filename: R2R PONE-D-20-21535.docx [file pone.0244695.s007.docx]

| **Reviewer’s Comment** | **Authors’ response/action** | **Edited Sections**  **(Page/Line)** |
| --- | --- | --- |
| **Editor’s comments** | | |
| Please ensure that your manuscript meets PLOS ONE's style requirements, including those for file naming. | We have revised the manuscript and formatted it according to the Instructions to Authors for PLOS ONE. |  |
| Include the pros and cons of the methods used by the cited papers that could influence or lead to mistaken conclusions | We have included the pros and cons of the methods in the context of the parasite detection methods used comparing them with more sensitive methods as well as variations in the sampling methods used in Table 1. |  |
| Please follow all the suggestions/recommendations given by the 2 reviewers | We have followed the suggestions and comments that have been made by the reviewers. The point-by-point responses to the commends raised have been done below. |  |
| **Reviewer 1: Major Comments** | | |
| Lines 71-72: Please clarify the basis for the Jan 1, 2000 – May 8, 2020 reckoning period | At the time of initial submission (July), the latest date for literature search was 8^th^ May 2020. However, we have now updated our search to include all articles published from 8^th^ May (which was our initial last day of literature search) to 30^th^ November. | Table 1  Line 75-76 |
| Lines 130-137: Please clarify if the studies mentioned conducted the diagnostic tests on PSAC at random or did the subjects exhibit clinical manifestations prior to testing. In other words, if possible, can you indicate in the table the sampling method for selecting the PSAC to be tested? | Most of the studies that have been included in our review were part of long terms programmes (longitudinal studies).. Most studies did not mention any clinical signs due to infection. Most of the sites or studies indexed infection among PreSAC based on infection in the school-aged children. Furthermore, for most studies, while the sampling methods were not directly mentioned, they established an exclusion and inclusion criteria for both villages and PreSAC and their mothers. | Table 1 |
| Lines 130-137: Could you also break down in terms of percentages and mention here the different diagnostic tests used? This could help qualify your statement on the discussion part on the possible higher infection rates in PSAC due to the low sensitivity of some of the tests, particularly Kato-Katz. | We have broken down the percentages for the main diagnostic tests. We have also edited the sentence to show that while Kato-Katz and Urine filtration technique were the main methods used, other methods were also used assupplements .The revised text is as follows:  *Kato Katz was the commonly used diagnostic test for screening for S. mansoni infection (96.6%, n=28/29) with one study using the Sodium acetate-acetic acid-formalin (SAF) solution concentration method alone. Other studies used a combination of Kato Katz with SAF (n=3), Kato Katz and point-of-care test circulating cathodic antigen (POC-CCA) in addition to Kato Katz (n=10), Kato Katz and enzyme-linked immunosorbent assays (ELISAs) (n=4) and real-time polymerase chain reaction (qPCR) (n=1). On the other hand, the urine filtration method was widely used among those who screened for S. haematobium (95.6%, 22/23) while one study used micro-hematuria in urine measured using urine reagent strips as a proxy-diagnosis for S. haematobium. Some studies (n=6) also used a combination of urine filtration methods and the dipstick assay for the diagnosis of S. haematobium (Table 1).* |  |
| Line 144: Provide relevant values for Central Africa for comparison with East Africa | The PPE value for Central Africa has been provided and the new sentence reads as “*Furthermore, stratification of infection by region showed that East Africa had a combined PPE of 27% (95% CI: 12–43) while only one study was obtained from Central Africa and had a PPE of 10% (95% CI: 0–27)* | Line 152-154 |
| Line 163: Please indicate the prevalence estimates for both species based on your results | The estimates for both species have been indicated. The updated sentence reads as….. *We observed that the prevalence estimates for S. mansoni ( IVhet PPE: 22% (95% CI: 9–36) were higher than for S. haematobium (IVhet PPE: 15%, 95% CI: 6–25).* | Line 173-174 |
| Line 176-177: Do the countries mentioned herein have MDA programs against schistosomiasis? If so, is it possible that these countries did not report including PSAC but could have done so in the past? | The countries mentioned have had MDA programmes but these were all tailored towards School-aged children; Namibia [1], Zambia [2-4] and Botswana [5-8].  Current evidence-based on these countries suggests that PreSAC have not been included in their studies. For instance, in Zambia, an earlier study, as well as other studies, were based on PSAC or adults. In Namibia (where the corresponding author is based), the last large scale study focusing on schistosomiasis was done in 2014 [1]. In Botswana, the last large scale chemotherapy programme was done between 1985–1993 [9]. Zambia has an NTD master plan (citation) while small scale MDA programmes were conducted in selected districts [3]. |  |
| Table 1: Under the column ‘Helminth,’ please write the scientific name properly. Write ‘S. mansoni’ and ‘S. haematobium’ instead. | The scientific names have been changed to read as suggested | Table 1 |
| **Minor Comments** | |  |
| Line 18: Remove ‘,’ after ‘including’ | The comma has been removed. The new sentence reads as” …….. including country and region where the study was done…..” | Line 18 |
| Line 19: Change ‘Schistosome’ to ‘schistosome | The word has been changed and the new sentence reads as “……*year and specific schistosome species observed*….” | Line 19 |
| Line 19: Change ‘specie’ to ‘species’ | An “s” has been added and the new sentence reads as “……*year and specific schistosome species*…..” | Line 19 |
| Line 46: Remove ‘ ” ’ afger ‘risks’ | The inverted comma has been removed and the new sentence reads……..*risk reduction and management of health risks*. | Line 47 |
| Line 58: Change ‘from school children’ to ‘SACs’ | School children have been changed to SAC and the new sentence reads as ……… *However, the success of school-based MDA programs is highly dependent on the number of* ***SAC*** *enrolled* | Line 56 |
| Line 59: Add ‘and’ after ‘(PSAC), | The word “and” has been added after PSAC. The new sentence reads as ……..*preschool-aged children (PreSAC),* ***and*** *certain adolescents and adults* | Line 61 |
| Line 61: Change ‘increase s’ to ‘increases’ | The correction has been made and the new sentence reads as ….. *This* ***increases*** *health inequalities and accumulation of potentially irreversible morbidities due* | Line 63 |
| Line 66: Does ‘SSA’ mean ‘Sub-Saharan Africa?’ If so, spell it out as it is mentioned for the first time here. | The word SSA has been written in full to sub-Saharan Africa. The new sentence reads as ……. *schistosomiasis infection among PreSAC in sub-Saharan Africa to illuminate the importance* | Line 69 |
| Line 80: Remove ‘,’ after ‘otherwise’ | The comma has been removed and the new sentence reads as…….. *Any disagreements were resolved by discussion unless otherwise arbitrated by the third author (MJC)* | Line 83 |
| Line 98: Add ‘,’ after ‘heterogeneity’ | A comma has been added after heterogeneity and the new sentence with a comma reads as……….. *the IVhet model regardless of heterogeneity, maintains a correct coverage probability at a lower detected variance* | Line 102 |
| Line 153: Add ‘,’ after ‘groups’ | A comma has been added after groups and the new sentence reads as……. *This contributes to the growing evidence that schistosomiasis infection in high endemicity settings cuts across all age groups, thus justifying……….* | Line 163 |
| Line 167: Change ‘result’ to ‘resulted’ | The word result has been changed to resulted and the new sentence reads as……… *According to these authors, this reduction could have resulted from MDA programmes* | Line 177 |
| Line 177: Add ‘,’ after ‘Zambia’ | A comma has been added and the new sentence reads as …….. *No studies focusing on PSAC were obtained from countries like Botswana, Lesotho, Namibia, and Zambia, thus reinforcing* | Line 188 |
| Line 183: Add ‘in’ after ‘intense’ | “In” has been added and the sentence reads as……. *This is because disease monitoring and surveillance are more intense in the SAC group* | Line183 |
| Line 199: Add ‘,’ after ‘sensitivity’ | A comma has been added and the new sentence reads as ……. *These methods have low sensitivity, thus affecting the detection of infection* | Line 210 |
| Table 1: Babatunde et al. Change ‘west Africa to ‘West Africa’ | “W” has been capitalized to read as West Africa | Table 1 |
|  |  |  |
| **Reviewer 2** | | |
| Line 27, What is "LFK"? Abbreviation should not be used in this point. | The initials have nee written in full and the sentence reads as …….. *A Luis Furuya–Kanamori index of 1.83 indicated a lack of publication bias* | Line 17 |
| Line 43 to 46, What are "Target 3.3", "Target 3.8" and "Target 3b"? It may be difficult to understand for readers unfamiliar to this scientific field. | These are targets that have been set by WHO to assist in the measurement of achievement of SDG 3. We have improved the sentence by making it clearer. The new sentence reads as ……. *Control of schistosomiasis is a fundamental part of the Sustainable Development Goals (SDGs) 3 which seeks to ensure healthy lives and promote well being for all ages with the health target 3.3 in part focusing on ending neglected tropical diseases by 2030, thus contributing to the achievement of universal health coverage (Health target 3.8). Achieving these targets in developing countries is dependent on supporting the research and development of vaccines and medicines, early warning, risk reduction and management of health risks (Target 3b)* [7]. | Line 43-48 |
| Line 62, Please correct "This increase s health" to "This increases health". | The correction has been made and the new sentence reads as ….. *This* ***increases*** *health inequalities and accumulation of potentially irreversible morbidities due* | Line 63 |
| Line 75, Please add double quotation marks to sub-Saharan Africa. | The quotation marks have been corrected and the new sentence reads as………. *The current study used the search terms “schistosomiasis AND pre-school OR "pre-school" OR "under five" AND "sub-Saharan Africa" NOT school-age children"* | Line 77-78 |
| Line 163, Author mentioned "prevalence estimates for S. haematobium were higher than for S. mansoni". Is it correct? Please check it. | We thank the reviewer for this comment. The typological error has been corrected and the new sentence reads as…… *prevalence estimates for S. mansoni ( IVhet PPE: 22% (95% CI: 9–36) were higher than for S. haematobium (IVhet PPE: 15%, 95% CI: 6–25).*.. | Line 173-174 |
| In Table 1, I think published year, sample size and prevalence of each paper should be added for easily understanding of tendency of Schistosoma prevalence | The year of publication for each paper, sample size and prevalence associated with each citation has been added. | Table 1 |

**References**

1.	Sousa-Figueiredo JC, Stanton MC, Katokele S, Arinaitwe M, Adriko M, Balfour L, et al. Mapping of schistosomiasis and soil-transmitted helminths in Namibia: the first large-scale protocol to formally include rapid diagnostic tests. PLoS neglected tropical diseases. 2015;9(7):e0003831.

2.	Simoonga C, Kazembe LN, Kristensen TK, Olsen A, Appleton CC, Mubita P, et al. The epidemiology and small-scale spatial heterogeneity of urinary schistosomiasis in Lusaka province, Zambia. Geospatial Health. 2008;3(1):57-67.

3.	Sokesi T, Malama K, Masaninga F, Vwalika B, Kachimba J, Mufunda J, et al. Lessons Learnt From the Implementation of Mass Drug Administration for Schistosomiasis and Soil-Transmitted Helminths in Lusaka Province, Zambia. Medical Journal of Zambia. 2016;43(2):82-7.

4.	Kalinda C, Mutengo M, Chimbari M. A meta-analysis of changes in schistosomiasis prevalence in Zambia: implications on the 2020 elimination target. Parasitology Research. 2020;119(1):1-20. doi: 10.1007/s00436-019-06534-0.

5.	Chimbari M. Key Public Health Issues in the Okavango Basin, Botswana. Harry Oppenheimer Okavango Research Centre, Maun, 2009.

6.	Geldenhuys P, Hallett A, Visser P, Malcolm A. Bilharzia survey in the eastern Caprivi, northern Bechuanaland and northern South West Africa. South African medical journal. 1967;41(31):767-71.

7.	Friis H, Byskov J. Schistosoma mansoni: intensity of infection and morbidity among schoolchildren in Matlapaneng, Ngamiland, Botswana. Tropical and geographical medicine. 1987;39(3):251-5.

8.	Friis H, Byskov J. The effect of praziquantel against Schistosoma mansoni-infections in Botswana. Tropical and geographical medicine. 1989;41(1):49-51.

9.	Mokgweetsinyana S, Ali M. Ngamiland Scistosomiasis Control Programme 1985-1993. In: Epidemiology Unit CHSD, editor.: Ministry of Health, Botswana; 2000.
